# Efficacy and Safety of Vitamin D Supplementation for Pulmonary Tuberculosis: A Systematic Review and Meta-analysis

**Published:** 2018-04

**Authors:** Ji WANG, Malong FENG, Shidong YING, Jianfang ZHOU, Xiaoqing LI

**Affiliations:** 1. Dept. of Infection, Ningbo Fenghua People’s Hospital, Zhejiang, China; 2. Dept. of Respiratory Disease, Ningbo Fenghua People’s Hospital, Zhejiang, China; 3. Dept. of Tuberculosis and AIDS, Ningbo Fenghua Disease Control and Prevention Center, Zhejiang, China; 4. Dept. of Hepatic Disease, Chongqing Traditional Chinese Medicine Hospital, Chongqing, China

**Keywords:** Vitamin D, Adjunctive therapy, Pulmonary tuberculosis (TB), Efficacy, Meta-analysis

## Abstract

**Background:**

Vitamin D might be promising to serve as an adjunctive therapy for pulmonary tuberculosis (TB). However, the results remained controversial. We conducted a systematic review and meta-analysis to evaluate the efficacy and safety of vitamin D in patients with pulmonary TB.

**Methods:**

Medline, SCOPUS, Google Scholar, EMBASE, Springer, and Science Direct were searched electronically from inception to Oct 2016. Randomized controlled trials (RCTs) and controlled clinical trials (CCTs) assessing the effect of vitamin D plus anti-tuberculosis treatment (ATT) versus placebo plus ATT on the treatment of pulmonary TB were included. Two investigators independently searched articles, extracted data, and assessed the quality of included studies. Data were analyzed using RevMan 5.3 software.

**Results:**

Five studies were included in this meta-analysis. Overall, compared with placebo intervention, vitamin D supplementation was found to have no significant effect on sputum smear negative conversion rates (RR=0.99; 95% CI=0.91 to 1.07; *P*=0.77), BMI (MD=0.11; 95% CI=−0.85 to 1.07; *P*=0.82) and ESR (MD=−2.29; 95% CI=−8.87 to 4.30; *P*=0.50).

**Conclusion:**

Vitamin D supplementation showed no influence on the improvement of sputum smear-negative conversion rates and BMI, as well as the decrease in ESR.

## Introduction

There has been 9.4 million tuberculosis (TB) that could result in 1.8 million deaths (1). TB has become an increasingly serious global public health problem and was regarded as a major cause of illness and death. TB treatment was challenging with the emergence of multidrug-resistant TB (MDR-TB) and extensively drug-resistant TB (XDR-TB) (1–6). However, there was still lack of ideal and available chemotherapies for the treatment of TB. It was urgent and valuable to develop novel anti-tuberculosis treatment (ATT) to control the global tuberculosis epidemic.

Vitamin D deficiency was widespread in active tuberculosis. Vitamin D deficiency was associated with an increased susceptibility to tuberculosis infection and increased the rate of conversion from latent to active tuberculosis (7–12). Thus, vitamin D might have the capability to decrease the risk of TB infection, prevent the progression from latent to active TB, decrease the duration and to improve treatment effectiveness as an adjunct to antimicrobial therapy (13, 14). Vitamin D had some potential in improving the anti-mycobacterial activity in monocytes, and it was partly mediated by the upregulation of the antimicrobial peptide LL-37 (15, 16). Vitamin D was found to increase the response to antimicrobial therapy for pulmonary TB (17).

Accumulating relevant RCTs showed that vitamin D supplementation failed to significantly improve sputum smear-negative conversion rates, and to reduce erythrocyte sedimentation rate (ESR) (18–21). Considering these inconsistent effects, we conducted a systematic review and meta-analysis to evaluate the efficacy and safety of vitamin D supplementation for the treatment of pulmonary TB.

## Methods

This review and meta-analysis was conducted according to the guidance of the Preferred Reporting Items for Systematic Reviews and Meta-analysis statement (22) and the Cochrane Handbook for Systematic Reviews of Interventions (23).

All analyses were based on previously published studies, and thus no ethical approval and patient consent were required.

### Literature search and selection criteria

Medline, SCOPUS, Google Scholar, EMBASE, Springer, and Science Direct were systematically searched from inception to Oct 2016, with the following keywords: vitamin D and pulmonary tuberculosis or pulmonary TB. No limitation was enhanced. To include additional eligible studies, the reference lists of retrieved studies and relevant reviews were also hand-searched and the process above was performed repeatedly until no further article was identified. Conference abstracts meeting the inclusion criteria were also included.

The inclusion criteria were as follows: study population, patients with pulmonary TB; intervention, vitamin D plus ATT; control, placebo plus ATT; outcome measure, sputum smear-negative conversion rates; and study design, randomized controlled trials (RCT) or controlled clinical trials (CCT).

### Data extraction and outcome measures

The following information was extracted for the included studies: first author, publication year, sample size, baseline characteristics of patients, intervention of vitamin D supplementation, intervention of control, study design, sputum smear-negative conversion rates, body mass index (BMI) and erythrocyte sedimentation rate (ESR). The author would be contacted to acquire the data when necessary.

The primary outcome was sputum smear-negative conversion rates. Secondary outcomes included BMI and ESR.

### Quality assessment in individual studies

Two reviewers independently performed data extraction and quality assessment. Four items were used to assess the quality of included studies based on Cochrane Collaboration recommended criteria: Adequate sequence generation, Allocation concealment, Blinding, and addressing the problem of incomplete outcome data.

### Statistical analysis

Mean differences (MDs) with 95% confidence intervals (CIs) for continuous outcomes (BMI, ESR, and serious adverse events) and relative risks (RRs) with 95% CIs for dichotomous outcomes (sputum smear-negative conversion rates) were used to estimate the pooled effects. The meta-analyses were performed using the fixed effect model or random effects model when appropriate. Heterogeneity was tested using the Cochran Q statistic (*P*<0.1) and quantified with the I^2^ statistic, which described the variation of effect size that was attributable to heterogeneity across studies. An I^2^ value greater than 50% indicated significant heterogeneity. Sensitivity analysis was performed to detect the influence of a single study on the overall estimate via omitting one study in turn when necessary. Owing to the limited number (<10) of included studies, publication bias was not assessed. *P*<0.05 in two-tailed tests was considered statistically significant. All statistical analyses were performed with Review Manager Version 5.3 (The Cochrane Collaboration, Software Update, Oxford, UK).

## Results

### Description of studies and quality assessment

**Fig. 1:**
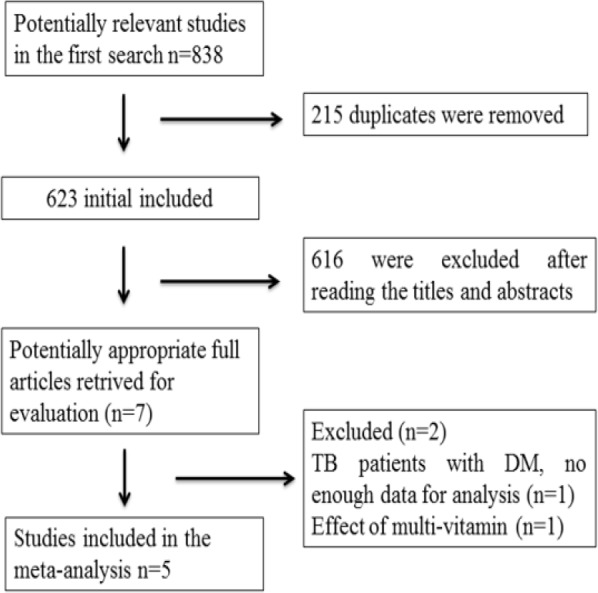
Flow diagram of study searching and selection process

Fig. 1 shows the search strategy and selection process of this meta-analysis. In all, 838 studies in the first search seemed to be potentially relevant. Overall, 215 duplicates were removed.

A total of 616 studies were excluded (irrelevant subjects) on the basis of initial screening of the titles and/or abstracts. One study was removed for not providing enough data of TB patients with diabetes mellitus (DM) for analysis and one study regarding the effect of multi-vitamin on pulmonary TB was removed. The remaining 5 articles were included in the meta-analysis (18–21, 24).

Five trials were conducted and they were all RCTs (18–21, 24). In four trials, high dose of vitamin D (50,000U–60,000U or 2.5 mg every 1–2 wk) was administrated orally or intramuscularly (Table 1). After contacting the authors, four articles got “yes” in “Adequate sequence generation”, “Allocation concealment” and “Blinding” (18–21). But one trial only got one “yes” in “Adequate sequence generation” (24) (Table 2).

**Table 1:** Characteristics of included studies

***NO.***	***Author***	***Vitamin D supplemented group***					***Vitamin D not supplemented group***				
		**No.**	**Age**	**Weight (kg)/BMI**	**Methods and Dosage**	**Follow up**	**No.**	**Age**	**Weight (kg)/BMI**	**Methods and Dosage**	**Follow up**
1	Kota 2011	15	38.4±19.6 (mean±SD)	49.1 ± 4.5 (mean±SD)	Oral calciferol (60,000 U/week) + ATT	12 w	15	40.2±17.7 (mean±SD)	44.6±5.6 (mean±SD)	ATT	12w
2	Martineau. 2011	62	30.7 (median)	20.1±3.1 (mean±SD)	Four oral doses of 2.5 mg vitamin D at 1, 2, 4 and 6 wk after the start of antimicrobial treatment+ ATT	8 w	64	30.5 (median)	20.2±2.7 (mean±SD)	placebo + ATT	8 w
3	Ralph 2013	101	29 (15–65) (median/range)	19.1 (13.3–32.5) (median/range)	Oral cholecalciferol 50,000 U (1250 mcg, 1 tablet) at baseline and on day 28+ ATT	24 w	99	26(15–73) (median/range)	19.3 (12.0–26.3) (median/range)	placebo + ATT	24 w
4	Salahuddin 2013	132	27.8±13.2 (mean±SD)	17.2 (11–25) (median/rang)	Intramuscular calciferol (60,000U/twice/month) + ATT	12 w	127	28.1±14.1 (mean±SD)	17.3 (11–27) (median/range)	placebo + ATT	12 w
5	Tukvadze 2015	100	32.4±10.6 (mean±SD)	-	50,000 U vitamin D3 orally 3 times for 8 consecutive weeks, followed by 50,000 IU vitamin D3 orally every 2 wk for an additional 8 wk+ ATT	16 w	99	34.1±12.4 (mean±SD)	-	placebo + ATT	16 w

**Table 2:** Quality assessment of included studies

***NO.***	***Included studies***	***Type of study***	***Adequate sequence generation***	***Allocation concealment***	***Blinding***	***Incomplete outcome data addressed***
1	Kota 2011	RCT	Y	U	U	N
2	Martineau 2011	RCT	Y	Y	Y	N
3	Ralph 2013	RCT	Y	Y	Y	N
4	Salahuddin 2013	RCT	Y	Y	Y	N
5	Tukvadze 2015	RCT	Y	Y	Y	N

RCT: randomized controlled trial, CCT: clinical controlled trial, Y: yes, N: no, U: unclear.

### Primary outcomes: Sputum smears negative conversion rates

**Fig. 2:**
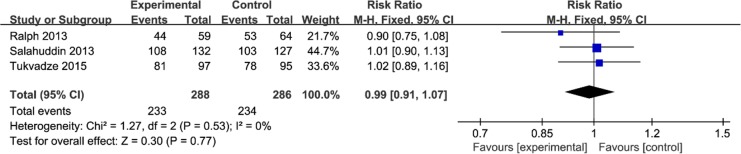
Forest plot for the meta-analysis of sputum smear-negative conversion rates

This outcome data were analyzed with the fixed-effect model, the pooled estimate of three included RCTs suggested that vitamin D supplementation had no influence on sputum smear-negative conversion rates (RR=0.99; 95% CI=0.91 to 1.07; *P*=0.77), with no heterogeneity among the studies (I^2^=0%, heterogeneity *P*=0.53, Fig. 2).

### Sensitivity analysis

No heterogeneity was observed among the included studies for the sputum smear-negative conversion rates. Thus, we did not perform sensitivity analysis by omitting one study in each turn to detect the source of heterogeneity.

### Secondary outcomes

**Fig. 3:**
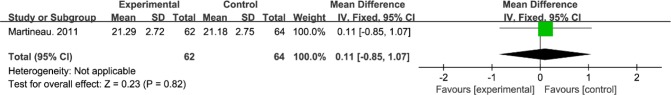
Forest plot for the meta-analysis of body mass index (BMI, kg/m^2^)

**Fig. 4:**
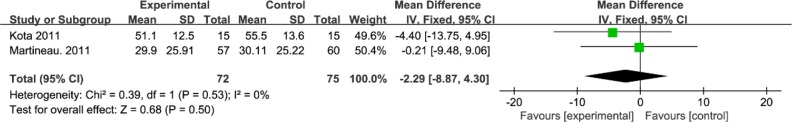
Forest plot for the meta-analysis of erythrocyte sedimentation rate (ESR, mm/h)

Compared to control group, vitamin D supplementation showed no significant influence on BMI (MD=0.11; 95% CI=−0.85 to 1.07; *P*=0.82; Fig. 3) and ESR (MD=−2.29; 95% CI=−8.87 to 4.30; *P*=0.50; Fig. 4).

### Adverse events

**Fig. 5:**
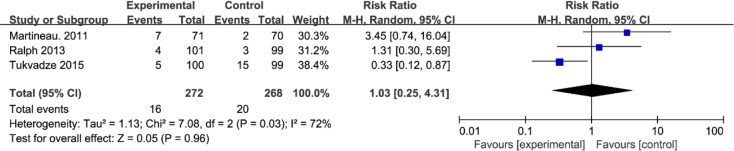
Forest plot for the meta-analysis of serious adverse events in TB patients

Serious adverse events were defined as any potentially life-threatening deterioration in health status within the study-monitoring period. They mainly included death, multi-organ failure, pneumonia, epistaxis and haemolytic anaemia, etc. There was no significant difference of serious adverse events between vitamin D supplementation group and placebo group (RR=1.03; 95% CI=0.25 to 4.31; *P*=0.96; Fig. 5).

## Discussion

Our meta-analysis clearly suggested that compared to placebo intervention, vitamin D supplementation had no significant influence on sputum smear-negative conversion rates, BMI and ESR. There were similar serious adverse events between vitamin D supplementation group and placebo group. This was the first meta-analysis to study the treatment efficacy of vitamin D supplementation for pulmonary TB.

Vitamin D could enhance the containment and killing of *Mycobacterium tuberculosis* through activating 25-hydroxyvitamin D receptors (VDRs) of immune cells (25). Patients with low 25-hydroxyvitamin D levels were found to have increased susceptibility to TB infection and showed high risk of progression from TB infection to disease (13, 16). 25-hydroxyvitamin D demonstrated some capability to induce mycobacterial killing (26). However, our meta-analysis showed that vitamin D supplementation had no substantial effect on the treatment efficacy of pulmonary TB.

These negative findings regarding the effect of vitamin D on pulmonary TB might be explained as follows: firstly, the “vitamin D deficiency” in patients with active TB and possibly not TB but other factors caused the deficiency of vitamin D. Secondly, there might be just some patients obtain substantial effects from a supplementary dose of vitamin D (21, 27). Thirdly, vitamin D as an adjunctive therapy might show some potential in treating pulmonary TB, but the dose and time period of use may be insufficient in current clinical studies.

Supplementary vitamin D 60000 IU was administered intramuscularly in 199 Pakistani pulmonary TB patients and resulted in significantly greater weight gain (1.14 kg) and greater radiological improvements compared with patients receiving placebo, but it showed no impact on sputum smear clearance rates (19). It was almost not possible to cause hypercalcemia in TB patients after using Vitamin D (28). Future studies should focus on higher doses of vitamin D, and targeted participant selection for active TB (29).

Several limitations should be taken into account. Firstly, our analysis was based on only five RCTs and more clinical trials with larger sample were needed to explore this issue. The follow-up period of some studies was not long enough to obtain the results of long-term effects and the follow-up time points varied across studies. Next, the data regarding radiological appearances, cavity closure rates and immunological indicators (e.g. IL-2, IFN-γ and TNF-α) were not tested in the included trials. Finally, some unpublished and missing data might lead bias to the pooled effect.

## Conclusion

Vitamin D supplementation showed no significantly favorable influence on improving outcome in patients with pulmonary TB, but more RCTs with higher dose of vitamin D and a longer time of use were needed to further confirm this issue for pulmonary TB.

## Ethical considerations

Ethical issues (Including plagiarism, informed consent, misconduct, data fabrication and/or falsification, double publication and/or submission, redundancy, etc.) have been completely observed by the authors.
